# Paclitaxel and Its Evolving Role in the Management of Ovarian Cancer

**DOI:** 10.1155/2015/413076

**Published:** 2015-06-07

**Authors:** Nirmala Chandralega Kampan, Mutsa Tatenda Madondo, Orla M. McNally, Michael Quinn, Magdalena Plebanski

**Affiliations:** ^1^Department of Immunology, Monash University, Level 6, The Alfred, Commercial Road, Melbourne, VIC 3181, Australia; ^2^Gynaeoncology Unit, Royal Women's Hospital, 20 Flemington Road, Parkville, Melbourne, VIC 3052, Australia; ^3^Department of Obstetrics and Gynaecology, University of Melbourne, Melbourne, VIC 3052, Australia

## Abstract

Paclitaxel, a class of taxane with microtubule stabilising ability, has remained with platinum based therapy, the standard care for primary ovarian cancer management. A deeper understanding of the immunological basis and other potential mechanisms of action together with new dosing schedules and/or routes of administration may potentiate its clinical benefit. Newer forms of taxanes, with better safety profiles and higher intratumoural cytotoxicity, have yet to demonstrate clinical superiority over the parent compound.

## 1. Introduction

Epithelial ovarian cancer is one of the leading causes of cancer death. One woman in 70 will develop ovarian cancer in her lifetime and the majority of these women will die from the disease. Although the prognosis for women with ovarian cancer is relatively poor due to its late presentation and the lack of scientifically validated screening tools, the 5-year survival rate increased significantly from 33% in 1982–1987 to 40% in 2000–2006 [1].

The standard of care in advanced epithelial ovarian cancer encompasses surgical staging and resection followed by administration of paclitaxel-platinum based chemotherapy. Maximal effort cytoreductive surgery, either initial or interval, with the aim of debulking to the point of no visible residual disease is associated with improved patient outcomes, with every 10% increase in the optimal cytoreduction rate leading to a 5.5% increase in median survival [2, 3].

Based on level-1 evidence, paclitaxel (175 mg/m^2^) in combination with carboplatin (AUC 5–7.5) every 3 weeks for 6 cycles, administered intravenously was accepted as the standard of care for first line chemotherapy by the GCIG Consensus Meeting in 2005 [4–8]. Approximately two-thirds of patients will respond to this combined surgery-chemotherapy approach, but tumour recurrence occurs in almost all these patients at a median of 15 months from initial diagnosis [9] and subsequent chemotherapy treatments are increasingly linked to chemoresistance. Response rates in this setting are proportional to the treatment-free interval. For instance, there is a 75% response when the treatment-free interval is at least two years but with a treatment-free interval of only six to nine months, the second line response is only 35% [10]. Other than its contribution as one of the first line agent in a combined therapy, paclitaxel has been promising, as early as 1990, as a second line agent in relapsed platinum-refractory epithelial ovarian cancer. The clinical therapeutic effect of paclitaxel is promising with modulation of dose and route of administration for use in advanced ovarian cancer, either in primary or recurrent setting.

There is thus still a strong need for novel, highly effective therapies for the treatment of advanced epithelial ovarian cancer. However, maximising the potential of paclitaxel is also a reasonable approach. This review will focus on how such new therapeutic strategies such as dose-dense paclitaxel chemotherapy, IP paclitaxel treatment, and newer forms of paclitaxel may add to the clinical benefit in patients with this malignancy.

## 2. The Evolution of Paclitaxel

**Figure 1:**
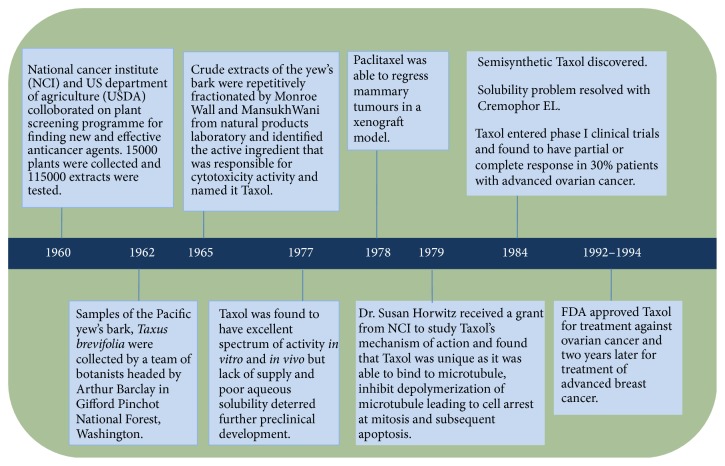
History of paclitaxel development. The search for the natural resources began in 1960 headed by NCI and USDA which led to discovery of Pacific yew's bark. The active ingredient was isolated by Monroe Wall and Mansukh Wani and was named Taxol. Taxol demonstrated both* in vivo* and* in vitro* antineoplastic activity, as well in xenograft models with breast tumours. The mechanism of action which was unique was identified and subsequently, after 22 years, Taxol entered clinical trials and demonstrated good cytotoxicity activity and was finally approved by FDA for treatment against ovarian and breast cancers.

Paclitaxel was not a chance discovery but the result of a collaborative effort of the National Cancer Institute (NCI) and the US Department of Agriculture (USDA) using a plant-screening programme in search of new and effective anticancer agents [26]. The history of paclitaxel development is summarised in Figure 1.

The NCI Plant Programme headed by Jonathan Hartwell, a natural product chemist, liaised with Robert Perdue, a USDA botanist and analysed over 15,000 natural plants worldwide as well as testing 115,000 extracts for anticancer activity over the period from 1960 to 1981. When the NCI screening programme concluded in 1981, paclitaxel was the only compound entered into clinical trials [27].

In August 1962, paclitaxel was isolated from the bark of the Pacific yew tree* Taxus brevifolia* Nutt. (Taxaceae) shipped in from Gifford Pinchot National Forest to USDA headquarters in Maryland by a 32-year-old USDA botanist, Arthur S. Barclay [26, 28, 29]. Although the cytotoxic activity of the bark of the samples soon became evident by September 1964 as it was found to be able to inhibit the* in vitro* growth in 9 KB cell cultures containing human oral epidermoid carcinoma [30, 31], it took Mansukh Wani and Monroe Wall, working under contract with the NCI at the Research Triangle Institute (Research Triangle Park, NC) till 1967 to isolate and identify the extract's most active ingredient and named it paclitaxel [26].

Paclitaxel was reported to have a broad spectrum of antitumour activity following* in vivo* screens in tumours implanted in laboratory mice [27]. With grant aid from NCI, Dr. Susan Horwitz from the Albert Einstein College of Medicine further enhanced the interest in paclitaxel with the novel discovery of its unique mechanism of action. Another breakthrough came in November 1978 with the ability of paclitaxel to cause tumour regression in a mammary tumour xenograft [26].

Although clinical trials to study the effect of paclitaxel on various cancers mushroomed, not many were able to commence as planned due to scarcity in the supply, the only source being the extremely slow-growing Pacific yew. Hartwell soon realized that for every 13 kilograms of dried bark, he was producing just half a gram of purified paclitaxel extract. In 1990, a petition to include the severely depleted* T. brevifolia* on the list of endangered species, concluded in the Pacific Yew Act being passed in 1992 to protect the tree [32].

Despite the scarcity, paclitaxel entered clinical trials after 22 years from its discovery in 1984. Wiernik and colleagues reported a phase 1 trial in 1987 [33]. Paclitaxel was found to have cytotoxic activity of clinical significance when a study on ovarian cancer concluded that 30% of patients with platinum-resistant ovarian cancer responded to paclitaxel therapy, either completely or partially [34]. Another issue was the insolubility of the paclitaxel in water but these problems were eventually overcome with a formulation in ethanol and Cremophor EL. Cremophor EL is a polyethoxylated castor oil, which was used as a vehicle for solubilisation of hydrophobic drug like paclitaxel [35]. However, the most serious side effect observed following use of Cremophor as the drug vehicle was that of hypersensitivity reactions, which were unpredictable and led to two deaths, and this almost halted any further clinical trials. A longer and slower infusion taking 24 hours reduced this severe adverse effect [33].

In December 1992, paclitaxel was registered as chemotherapy for the treatment for ovarian cancer. To meet increasing demand, a commercially viable semisynthetic was developed by Robert Holton and colleagues [31]. Researchers also tested the effectiveness of paclitaxel as a treatment for advanced breast cancer. Subsequent clinical trials found that the drug was effective against this disease, and, in 1994, the FDA approved paclitaxel for use against breast cancer. Paclitaxel is now used (either as a single agent or in combination with other drugs such as cisplatin or carboplatin) for the treatment of ovarian cancer [36], breast cancer [5], and non-small-cell lung cancer [37].

## 3. Chemical Structure

**Figure 2:**
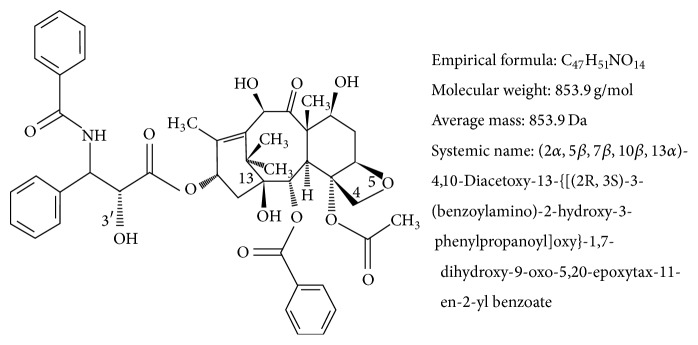
Chemical structure of paclitaxel. Paclitaxel consist of taxane ring with a four-membered oxetane side ring at positions C4 and C5 and an active homochiral ester side chain at C13 that binds to microtubules in a guanosine triphosphate (GTP) independent manner to induce cytotoxicity activity.

Paclitaxel, a class of taxane drugs, is a diterpenoid pseudoalkaloid with the empirical formula C_47_H_51_NO_14_ (Figure 2) and has a corresponding molecular weight of 853.9 g/mol. Paclitaxel consist of two molecules: a taxane ring with a four-membered oxetane side ring at positions C4 and C5 and a homochiral ester side chain at C13. The side chain at C13 plays a crucial role, as this is the active portion that binds to microtubules, stabilises the tubulin bundles, and stimulates disassembly of microtubules in a guanosine triphosphate (GTP)-independent manner. As a result, cell proliferation is inhibited by halting the cell cycle at the metaphase/anaphase boundary and by formation of an incomplete metaphase plate of chromosomes, induced by the stabilization of the microtubule dynamics. Extensive research has concluded that an intact taxane ring and an ester side-chain were essential for cytotoxic activity [38, 39].

## 4. Mechanism of Action

Paclitaxel is an anticancer drug that targets microtubules. Microtubules consist of cylindrical hollow bodies of about 25–30 nm in diameter, composed of polymers of tubulin in dynamic equilibrium with tubulin heterodimers (consisting of alpha and beta protein subunits) [40, 41]. The principal function of microtubules is the formation of the mitotic spindle during cell division. In addition, they are required for the maintenance of cell structure, motility, and cytoplasmic movement within the cell. The synthesis of tubulin and the assembly of microtubules occur during the G2 phase and the prophase of mitosis. Microtubules are in a state of dynamic equilibrium with their subunit tubulins *α* and *β*, arranged in a head to tail fashion, with preferential faster growth (plus ends) at one end, and slower growth (minus ends) at the other end. Under steady-state conditions, the length of the microtubule is unchanged, as the net tubulin assembly rate equals the net disassembly rate. The minus ends part of microtubule are usually anchored mainly at the centrosome [42], while the plus ends explore the cytoplasm and interact with cellular structures [43, 44].

Dr. Horwitz discovered that paclitaxel, unlike vinca alkaloids which prevent microtubule assembly, prevents cell division by promoting the assembly of stable microtubules especially from *β*-tubulin heterodimers and inhibits their depolymerisation; hence, exposed cells are arrested in the G2/M-phase of the cell cycle [45] and eventually undergo apoptosis [46], thereby inhibiting cell replication [40, 47]. Paclitaxel binds specifically in a reversible manner to the N-terminal 31 amino acids of the beta-tubulin subunit in the microtubules rather than to tubulin dimers [47, 48]. Paclitaxel also has the unique ability to promote microtubule formation* in vitro* even at cold temperatures (4°C) and in the absence of GTP [46].

Further research into understanding the molecular mechanisms of microtubule formation has discovered that paclitaxel may have other mechanism that allows cells to escape drug toxicity, which then develops into resistance and failure in chemotherapy [44]. Ganguly and his colleagues found that microtubule dynamics suppression was not related to cell division. Treatment of mutant cell lines, Tax 11-6 (mutation in *α*-tubulin) and Tax 18 (mutation in *β*-tubulin) at concentration of 50 to 100 nmol/L that should hypothetically increase microtubule assembly to more normal levels and allow the cells to proliferate normally, further suppress microtubule dynamics rather than restoring behaviour to normal [44]. Low concentrations of paclitaxel that suppressed dynamics; hence, they did not affect the rate of microtubule detachment; but the higher drug concentrations that allowed normal cell division strongly inhibited detachment in the mutant cell lines and returned the rate to near normal levels. This action seems to be related to ability of paclitaxel to inhibit microtubule fragmentation rather than its ability to suppress microtubule dynamics, which was further confirmed by live cell imaging that demonstrated that microtubule detachment from centrosomes might be responsible for generating microtubule fragments, a process that was reversed by paclitaxel [44]. Recent studies have also found that at low concentrations (less than nanomolar concentrations), paclitaxel inhibits the depolymerisation of microtubules, whereas at high-dose, paclitaxel increase the number and mass of microtubules, hence increasing stability of microtubules and also render them nonfunctional by blocking the detachment of microtubule minus ends from centrosomes rather than plus ends [44, 49].

The proposed mechanism of action of weekly paclitaxel administration may be based on its apoptotic effects. The induction of apoptotic modulator genes by paclitaxel appears to be independent of microtubule stabilization. It may be due to modulation of the transcription of different genes, such as DNA-damage response proteins, cytokines or proteins involved in the control of cellular proliferation, apoptosis, and inflammation. The apoptotic effect of paclitaxel is dependent on the concentration and duration of exposure. With concentration amounts of at least 10 nM and exposure of at least 12 hours, apoptosis may be induced in the S phase without mitotic arrest. Two different mechanisms of apoptosis have been proposed, depending on the dose concentration of paclitaxel. At concentration of ≥9 nM, paclitaxel induces Raf-1 activation, which is responsible for apoptotic control. At ≤9 nM, there is absence of Raf-1 kinase involvement, but apoptosis induction still occurs under the influence of p53 and p21 [50, 51]. With the same concentration at 24-hour exposure, in addition to apoptotic arrest, paclitaxel produces an irreversible mitotic arrest [52]. p53 is a major tumour suppressor, regulating proliferation and apoptosis, and its mutation occurs in more than 50% of human cancers. In normal cells, DNA damage increases p53 levels, which then activates a G1 cell-cycle arrest mediated by p21, to promote either the DNA damage repair mechanisms or apoptosis and naturally limiting proliferation of genetically transformed cell clones. A functional p53 signaling pathway is necessary to sensitise cancer cells to DNA-damaging chemotherapeutic agents to reduce chemo resistance. Currently, paclitaxel activity is considered to be independent of p53 status, which is an important aspect as the presence of silent or mutated p53 does not modify the sensitivity of cancer cells to taxane, thus reducing chemoresistance [53–56].

Paclitaxel also exerts its mechanism of action by activation of multiple signal-transduction pathways, which may be associated with proapoptotic signaling. The pathways associated with paclitaxel are TLR-4 dependent pathway (either via MyD88 dependent or independent pathway), c-Jun N-terminal kinase (JNK), P38 Mitogen activated protein (MAP) Kinase, nuclear factor kappa B (NF-*κ*B), Janus kinase- (JAK-) signal transducer and activator of transcription factor (STAT) pathway. One of the pathways for induction of apoptosis is via mitogen-activated protein kinase (MAPK) pathway, resulting in dephosphorylation of the proapoptotic protein Bad and Bax, phosphorylation of Bcl2, and induction of apoptosis. Bad and Bax (promote apoptosis) and Bcl2 (suppress apoptosis) are regulatory proteins which are members of the Bcl2 family and are involved in programmed cell death. Induction of cytokines and pro-inflammatory proteins will lead to immunomodulatory effect of paclitaxel at low-dose concentration and cell death at higher dose. Changes that occur in these pathways are also responsible for development of resistance to paclitaxel [57–60].

Weekly paclitaxel also exhibits strong angiogenic inhibitory activity [61, 62]. In murine studies, paclitaxel was able to reduce new vessel formation at low, noncytotoxic doses (0.3 and 6 mg/kg per day in mice) by suppressing VEGF expression [63, 64]. Low dose, weekly paclitaxel has been tested in patients with advanced ovarian cancer, metastatic melanoma, and advanced head and neck cancer resulting in stable disease [65–67]. This additional mechanism of action of paclitaxel by rescheduling the duration of administration may be helpful in cancers, which have become resistant to the same drug given on a 3-weekly conventional schedule [11, 13].

**Figure 3:**
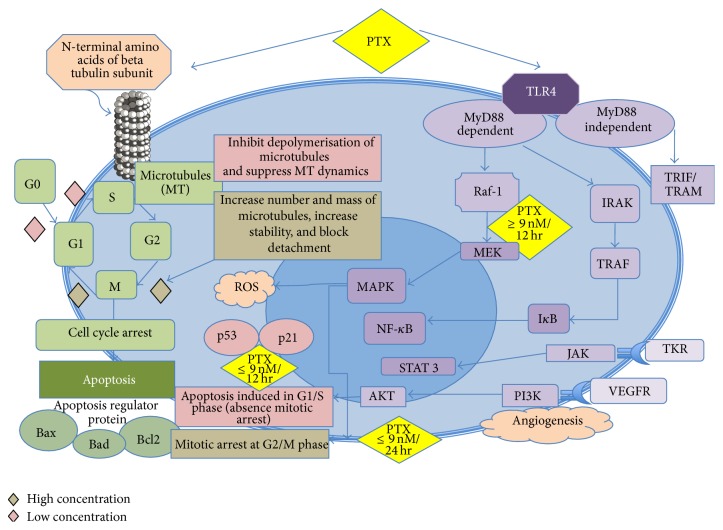
Mechanism of action of paclitaxel. Paclitaxel targets microtubules. At high concentration, PTX causes mitotic arrest at G2/M phase whereas at low concentration, apoptosis is induced at G0 and G1/S phase either via Raf-1 kinase activation or p53/p21 depending on the dose concentration. Even at lower dose but with exposure beyond 24 hours, paclitaxel can cause mitotic arrest. Paclitaxel also activates multiple signaling pathway to exert proapoptotic activity as well as immunomodulatory effect. Paclitaxel also develops resistance via these signaling pathways. PTX: Paclitaxel, TLR4: Toll-like receptor 4, G0: resting phase, G1; cells enlarge and make new protein, S phase: DNA replication, G2: preparation for division, M phase: cell division/mitosis, Raf-1: Raf kinase family, MEK/MAPK: mitogen activated protein kinase, IRAK: IL-1 receptor associated kinase, TRAF: TNFR associated factor, NF-*κ*B: nuclear factor kappa B, TRIF/TRAM: TIR-domain-containing adapter-inducing interferon-*β*, TKR: tyrosine kinase receptor, VEGFR: vascular endothelial growth receptor, PI3K: phosphoinositide 3-kinase, JAK: janus kinase, STAT: signal transducer and activator of transcription factor.

Paclitaxel had also been reported to induce reactive oxygen species (ROS) generation and increase hydroperoxide production by enhancing the activity of nicotinamide adenine dinucleotide phosphate (NADPH) oxidase, which contributes to oxidative stress and may play a role in the potency of the anticancer activity of paclitaxel [68, 69]. The combination of paclitaxel with inhibitors of glucose (i.e., 2-deoxy-D-glucose, 2DG) and hydroperoxide (i.e., L-buthionine-S, R-sulfoximine, BSO) metabolism has been found to selectively enhance breast cancer cell killing via hydrogen peroxide-induced metabolic oxidative stress, which may be effectively utilised to treat breast cancers [69]. However, the relationship of oxidative stress to the overall cytotoxicity mechanism of paclitaxel is not well established. The mechanism of action of paclitaxel is summarised in Figure 3.

## 5. Mechanism of Drug Resistance 

The mechanism of paclitaxel resistance is complex, involving multistep and multiple genes, and not yet fully elucidated. Development of drug resistance, leading to aggressive disease that is refractory to treatment, is responsible for 90% of the deaths among patients with advanced ovarian cancer [70]. Multidrug resistance (MDR) is a phenomenon defined by the ability of cancer cell to overcome either structurally or functionally unrelated cytotoxic drugs. Mechanism of MDR can be broadly divided into three types: (a) external mechanism involving changes in pharmacokinetics of drug or (b) within tumour microenvironment which includes hypoxia leading to clonal selection pressure or (c) at cellular level [71]. MDR can be intrinsic or acquired. Intrinsic MDR or also known as inherent occurs when cancer cells develop resistant to any chemotherapy and this is most likely attributed by tumour microenvironment. Acquired MDR develops following exposure to chemotherapeutic agents and may occur as a result of the pharmacokinetic of drug itself or secondary to changes within the tumour cells [71].

Paclitaxel-derived resistance is mainly attributed by changes involving mRNA and protein (such as multiple ribosomal genes and translation factors) synthesis, oxidative stress (UGT1A6, MAOA, and CYBA), glycolysis (ADH1A, HK1, and ENO3), glutathione metabolism, and leukocyte transendothelial migration pathways [71]. A study on gene expression changes on a series of drug resistant ovarian cancer cell lines following exposure to paclitaxel, cisplatin, or doxorubicin revealed that, among 845 genes analysed, a total of 337 genes were significantly altered in cells resistant to paclitaxel [70]. Changes in genes expression may create interpatient variation in drug effect, alteration in tumour microenvironment, and changes within cellular structures, metabolism and functions that promotes towards drug resistance.

Tumour cells grow rapidly with formation of numerous new vessels attributing to irregular blood flow and increased oxygen demand which lead to development areas devoid of adequate oxygenation within ovarian cancer tissues [72]. In response to chronic hypoxia (oxygen depletion), tumour microenvironment adapts processes to keep cells alive in hypoxic and acidic state. This hypoxic adaptation promotes tumour proliferation, dissemination, and progression of disease [73] and also reduces chemosensitivity [74].

Hypoxia-induced chemoresistance occurs due to genomic instability. Oxygen depletion induces proteomic and genomic changes that activates the level of p53, inhibits apoptosis, promotes angiogenesis (angiogenic molecules such as vascular endothelial growth factor [VEGF] and angiogenin), upregulates growth factors (platelet-derived growth factor [PDGF], transforming growth factor-beta and insulin-like growth factor [IGF]), and induces anaerobic metabolism and glycolysis (glycolytic enzymes, glucose transporters) leading to reduction in potential for cell cycle arrest and cellular differentiation which prevent tumour cell death [74]. Hypoxia can induce either p-53-dependent (involving Apaf-1 and caspase-9 effector pathways) or p53-independent (involving hypoxic-inducible factor 1 [HIF-1] and Bcl-2 family genes pathways) apoptosis in both normal and tumour cells. HIF-1 is a common transcription factor, consisting of HIF-1*α* and HIF-1*β* subunits, which controls hypoxic-inducible genes, and the concentration of HIF-1 protein increased exponentially in the presence of hypoxic environment. Loss of apoptotic potential of tumour cells mediated by hypoxic environment and HIF-1, which arrest cell cycle at G0/G1, reduces chemosensitivity to chemotherapeutic agents including paclitaxel [70, 75].

Other transcriptional factors, which are activated by hypoxia, are nuclear factor kappa B (NF-*κ*B) and STAT 3 also known as proinflammatory transcriptional factors. NF-*κ*B and STAT 3 regulate multiple gene products, which are involved in inflammation, angiogenesis, cell survival, proliferation, and metastasis. Paclitaxel, which mediates its cytotoxic action via NF-*κ*B pathway, is vulnerable to develop resistance in the presence of hypoxic tumour microenvironment, with activation of NF-*κ*B transcription factor, which induces serine phosphorylation and also regulates BCL-2, an antiapoptotic protein, preventing cell death and promoting tumorigenesis [76]. Inhibition of activated STAT 3 found overexpressed in most paclitaxel-resistant ovarian cancer cells, has resulted in reduction in paclitaxel resistance. However, further research is required to determine the exact mechanism [77].

Intracellular drug concentration is important for effective cytotoxic activity of paclitaxel. Drug resistance may be attributed to either reduced accumulation of drug within ovarian tumours or increased efflux of drug from tumour cells [78]. Alteration in pharmacokinetic of paclitaxel either attributed by the drug itself such as lower concentration of drug administered, shorter duration of drug exposure, high first pass metabolism, increased hepatic or renal clearance, inadequate binding to tubulins, microtubules or other macromolecules, or presence of higher tumour density or vascularity may result in reduced accumulation of intratumoral drug concentration [71].

Drug efflux is the most common mechanism for chemoresistance observed in paclitaxel-treated cancer cells. Drug efflux from cancer cells is mediated by ATP-binding cassette (ABC) transporters such as P-glycoprotein (P-gp). P-gp also known as ABCB1 or multidrug-resistance associated-protein (MRP), a transmembrane protein, encoded by MDR1 gene, plays a role as an efflux pump which is crucial for occurrence of many cellular processes that require transfer of substrates across cellular membranes. P-gp hence can affect intracellular drug concentration and have been shown to correlate,* in vitro*, to chemosensitivity to paclitaxel [59]. However, evidence for their role in clinical drug resistance in ovarian cancer has emerged with P-gp found to be overexpressed in ovarian cancer cells* in vitro* and also on paclitaxel-resistant cell lines [70]. P-gp and MDR1 expression levels were higher in chemoresistant ovarian cancer patients as compared with their chemosensitive counterpart. Overall survival time was also statistically higher in patients with low expression of P-gp and MDR1 in their tumor tissues. Hence, P-gp and MDR1 may have a predictive role in determining the outcome of patients with advanced ovarian cancer [58, 79–82]. Mechanism of paclitaxel resistance is complicated and specifically inhibiting ABC transporters have given mixed results [59, 83].

Another major mechanism of paclitaxel resistance is mediated by either a reduction in total intracellular tubulin concentrations, point mutation at prominently expressed tubulin genes, or by selective alterations in expression of tubulin isotypes, such as Class III *β*-tubulin [52, 84, 85]. Microtubule dynamic stability was significantly impaired in the paclitaxel-resistant cells [86]. Microtubule dynamics disruption caused by paclitaxel result in cell cycle arrest, apoptosis, and drug resistance.

Other mechanism responsible for development of paclitaxel resistant are inhibition of apoptosis, activation of mitogen-activated protein kinase, Raf-1 kinase or intracellular signalling pathway PIK3 (phosphatidylinositol-4,5-bisphosphate 3-kinase), changes in apoptotic regulatory proteins such as Bcl-2, increase in expression of proinflammatory cytokines such as TNF-*α*, IL-6, and IL-8, and activation of lipopolysaccharide-inducible genes and tumour suppression protein p53 [57, 83, 86, 87].

In summary, paclitaxel resistance is multifactorial, including changes in signaling pathways, upregulation of P-glycoprotein (P-gp) [80, 88, 89], alteration in tubulin dynamic, mutations in *β*-tubulin gene or expression of *β*-tubulin isotypes [52, 84, 85], and changes in apoptotic mechanism. Identification of mechanism of resistance could help to potentially develop novel agents to improve chemosensitivity of patients with advanced ovarian cancer.

## 6. Pharmacokinetics

Paclitaxel is a white to off-white crystalline powder, highly lipophilic, and is insoluble in water and so difficult to formulate into solution. It has a melting point of 216-217°C [90, 91]. More than 90% of the drug binds rapidly and extensively to plasma proteins [33, 40], whereas binding onto red blood cells is approximately 50%. The presence of pre,medication drugs (before chemotherapy) such as ranitidine, dexamethasone, or diphenhydramine did not affect protein binding of paclitaxel. Paclitaxel has a large volume of distribution of about 55 L/m^2^ but this is reduced in the females [33].

**Figure 4:**
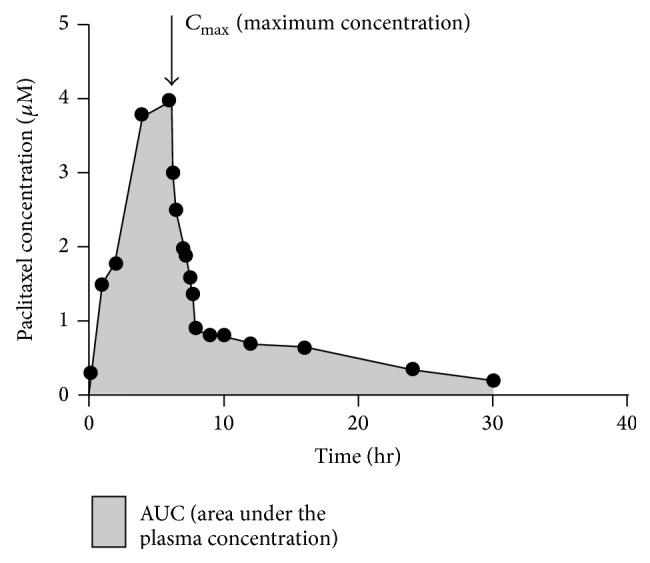
Plasma pharmacokinetics of paclitaxel. The pharmacokinetics generally were linear for 6 or 24 hour infusions but become nonlinear for infusions of shorter durations due to variation in the elimination clearance with the dose administered. An increase in plasma concentration of paclitaxel, results in disproportionate larger increase in *C*_max⁡_ (maximum plasma concentration) and AUC (area under plasma concentration), followed by decrease in drug elimination from body tissues.

The disappearance of paclitaxel from plasma is traditionally believed to be biphasic [33]. The initial rapid decline represents distribution to the peripheral compartment and elimination of the drug. The later phase is due, in part, to the slow efflux of paclitaxel from the peripheral compartment [92]. In the presence of more sensitive assay methods and later sampling times, a three-compartment model seems to be more accurate [40, 93]. Paclitaxel has been shown to have a nonlinear pattern of pharmacokinetics (Figure 4). The pharmacokinetics were generally linear for 6- or 24-hour infusions, but become nonlinear for infusions of shorter durations because the elimination clearance varies with the dose administered. With increasing plasma concentration of paclitaxel, there is a disproportionately larger increase in *C*_max⁡_ (maximum plasma concentration) and AUC (area under plasma concentration), accompanied by decrease in drug elimination from body tissues. The clinical importance of this nonlinear pattern is that dose escalation may result in a disproportionate increase in toxicity, whereas dose reduction may affect its efficacy [33, 90, 91, 94].

The pharmacokinetics of paclitaxel has been evaluated over a wide range of doses, up to 300 mg/m^2^ and with infusion schedules ranging from 3 to 24 hours. Maximum plasma concentrations are dose-related. In patients treated with single dose infusion of 135 and 175 mg/m^2^ given as 3- and 24-hour infusions, mean steady state volume of distribution has ranged from 198 to 688 L/m^2^, indicating extensive extravascular distribution and/or tissue binding. Terminal half-life has ranged from 1.3 to 8.6 hours (mean 5 hours) [40, 93], and total body clearance has ranged from 11.6 to 24.0 L/hr/m^2^. Preclinical results in animals have shown high levels in most tissues. Being highly protein-bound, paclitaxel has a high affinity for distribution in specific tissues including kidney, lung, spleen, and extracellular fluids like ascites and pleural fluids [40, 95] but the uptake of the drug in the brain is minimal [96, 97]. Exposure to paclitaxel is relatively high in tumour tissue compared with other tissues, and in addition to slow elimination from tumour tissue, the AUC in tumour tissue is about five-fold higher than that in plasma [97].

There is no evidence of accumulation of paclitaxel with multiple treatment courses as the variability in systemic paclitaxel exposure, as measured by AUC (0–∞) (area under plasma concentration-time curve from time 0 to infinity) for successive treatment courses, are minimal [98]. Paclitaxel clearance is however sequence dependent. Patients receiving a platinum-based agent prior to paclitaxel have lower clearance and greater clinical toxicity than patients receiving paclitaxel before cisplatin [93].

The drug, administered intravenously, undergoes an extensive P-450 mediated hepatic metabolism by cytochrome enzymes (CYP3A and CYP2C8), with 70–80% being excreted into bile by adenosine triphosphate- (ATP-) binding cassette multidrug transporters such as P-glycoprotein (P-gp) and multidrug resistance protein 2 (MRP-2), either as metabolites or as the parent drug. Variation in MRP-2 activity has been found to have direct effect on the effective exposure to paclitaxel [99]. The bioavailability is poor following oral administration due to enterocyte expression of P-gp and first-pass metabolism in the liver. Most of the drug is eliminated in feces. Less than 10% drug in the unchanged form is excreted in the urine, indicating extensive nonrenal clearance [96]. Combinations of inhibitors of CYP3A and P-gp might possibly improve the oral bioavailability of the taxanes [89, 97, 100].

## 7. Paclitaxel and the Immune System

The immune system consists of two different, but interacting mechanisms, which are innate and adaptive mechanisms. The innate mechanism is the first line of defense and consists of a group of cells which includes mainly macrophages, dendritic cells (DCs), and natural killer (NK) cells while the adaptive immune mechanism is represented by T-lymphocytes. All the components of innate and adaptive mechanism are involved in antitumour immunity, which includes tumour recognition, control, and elimination. However, cancer cells are able to evade immune detection and actively suppress antitumour immunity.

**Figure 5:**
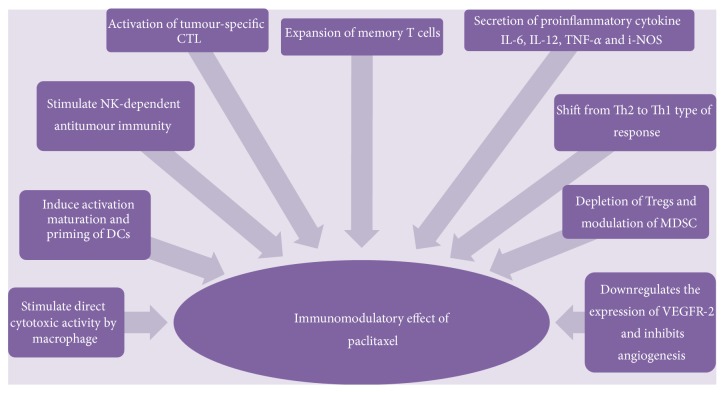
Immunomodulatory effect of paclitaxel. Paclitaxel, a toll-like receptor 4 (TLR4) ligand binds to TLR4 receptor and triggers TLR4 signaling via MyD88 dependent and independent pathway. Paclitaxel then promotes anticancer immune response directly by stimulating macrophages to kill cancer cells or indirectly by secretion of proinflammatory cytokines which upregulates activation of DCs, NK and tumour specific CTL. Paclitaxel promotes effective CTL response by upregulation of mannose-6-phosphate which facilitate permeability to granzyme B and cytokine patterns of T helper type 1. Paclitaxel modulates MDSC and ablates Tregs. Memory T cells (CD4+CD45RO+ and CD8+CD45RO+) increased significantly while regulatory T cells (Tregs) decreased around 2 weeks, creating an opportunity window for dose-dense therapy and immunomodulatory agents to achieve clinical benefit. Dose-dense and low dose paclitaxel also blocks new vessel formation by downregulating VEGF-receptor 2, reduces resistance by alternative mechanism of action.

Paclitaxel has a wide range of dose dependent immunomodulatory effects described in Figure 5. At standard dose, paclitaxel is broadly immunosuppressive and this is because it inhibits a number of cell types involved in tumour rejection such as macrophages, effector T cells and NK cells [101]. However, at lower doses than typically used for chemotherapy, paclitaxel has an important immunogenic role, promoting antitumour immunity [101, 102]. Hence, understanding the immunological basis of paclitaxel may help to maximise the manipulation of the immunomodulatory effects of paclitaxel to develop better therapeutic options in the management of advanced ovarian cancer.

Paclitaxel is a ligand to toll-like receptor 4 (TLR4), which is expressed on innate immune cells including macrophages. The binding of paclitaxel to TLR4 on macrophages triggers the MyD88, mitogen-activated protein kinase (MAPK) and transcription of nuclear factor NF-kappa B (NF-*κ*B) pathways (Figure 3) resulting in their activation and release of immunostimulatory cytokines such as tumour necrosis factor-*α* (TNF-*α*), interleukin-1 (IL-1), interleukin-6 (IL-6), and interleukin-8 (IL-8) [103–105]. Activated macrophages can either cause direct tumour cell lysis via release of lysosomal enzymes and nitric oxide (NO) or indirectly by activating NK cells, DCs, and tumour-specific cytotoxic T-lymphocytes (CTLs) [101].

NK cells are also innate immune cells capable of killing tumour cells, particularly those cells that have reduced class I MHC expression and therefore can escape killing by CTLs. Paclitaxel can enhance NK cell function by inducing mRNA and protein production of perforin, an effector molecule involved in NK cell-mediated cytotoxicity [106]. However, the effect of low dose paclitaxel on NK cells is controversial. In another study it was found that treatment of human NK cells with paclitaxel effectively inhibits NK cell-mediated killing of K562 (human erythroleukemia) target cells* in vitro* without affecting the viability of NK cells [107]. However, weekly paclitaxel therapy for non-small cell lung cancer patients was only able to reduce the NK cell function up to completion of the first cycle, but thereafter it gradually recovered [108].

Dendritic cells also express TLR4 and can be directly activated by paclitaxel. The binding of paclitaxel to TLR on immature DCs, promotes DC maturation by upregulating antigen-processing machinery gene components, costimulatory molecules, and IL-12p70 [102]. This enhances the ability of DCs to prime T cells, which facilitate antitumour immunity [109]. Paclitaxel can also promote cytotoxic T-cell function by upregulating mannose-6-phosphate which facilitates permeability to granzyme B and also induces cytokine production patterns typical of the T helper type 1 phenotype, via IL-2 (CD4 T cell) and IFN-*γ* (CD8 T cell) secretion [104, 110]. Activated CD8+ T cells can differentiate into CTL type 1 (Tc1) cells, producing mainly IFN-*γ*, and CTL type 2 (Tc2) cells, producing mostly IL-4, IL-5, and IL-10. Tc1 cells are potent CTL involved in the defense against cancer. Tc1 cells are able to recognise tumour antigens in the context of MHC class I and mediate tumour cell lysis. The role of Tc2 cells in the immune response is not clearly known, although their presence has been associated with disease progression [111].

CD4+ CD25+ regulatory T-cells (Tregs), constituting approximately 5–10% of peripheral CD4+ T cells [112, 113] play an important role in tumour immune evasion [114]. There is growing evidence that supports the existence of elevated numbers of Tregs in tumours [115–117], which may suppress immune responses of other CD4+ and CD8+ cells [118–120] and promote tumour progression. In murine studies, paclitaxel exhibits the ability to reduce the number and size of Treg cells thereby enhancing antitumour immunity [119, 121]. Similar findings were seen with a selective reduction in the size and number of Treg populations in peripheral blood samples from non-small cell lung cancer (NSCLC) patients [122].

Paclitaxel also decreased the expression of the antiapoptotic molecule Bcl-2 in Treg cells while increasing the corresponding proapoptotic member Bax, which contributed to the apoptotic sensitivity of Treg to paclitaxel [87]. This was seen in a study using the 3LL Lewis tumour model, which demonstrated that Treg cells exposed to paclitaxel displayed down regulation of Bcl-2 and upregulation of Bax, and upon blocking the Bcl-2 pathway, the ability of paclitaxel to ablate Treg cells compared to T effectors was impaired and both Treg and T effectors were affected. These results may suggest that paclitaxel targets Bcl-2 rather than tubulin to contribute to the distinctive effect on Treg cells [119]. It has recently been shown* in vitro* that paclitaxel could target Bcl-2 and therefore lead to apoptosis. Bcl-2 family proteins are key regulators of apoptosis and play an important role in tumourigenesis and multi-drug resistance by blocking apoptosis [119, 123].

Few studies have also shown effect of paclitaxel on myeloid-derived suppressor cells (MDSCs), a heterogeneous population of immature myeloid cells, immunosuppressive in nature are recruited by cancer cells and are found in increased amount in advanced epithelial ovarian cancer [124]. In a murine study using C57BL/6 mice, administration of paclitaxel at ultralow dose modulates MDSCs to differentiate into DCs in a TLR4 independent manner [110]. Hence, paclitaxel in ultralow, noncytotoxic doses together with its angiogenesis blocking ability may potentially enhance the efficacy of immunotherapy by ablation of immunosuppressive populations such as Tregs and MDSCs in tumour-bearing hosts [50, 102].

In immunocompromised patients with advanced ovarian cancer, with increased Tregs and decreased levels of CD4+ T cell, CD8+ T cell, and NK subsets, after a single course of combined carboplatin and paclitaxel chemotherapy, the immunosuppression is reversed around 12 to 14 days later, but not before one week or after three to four weeks. Not only are Tregs decreased, but also the proportions of IFN-*γ* secreting CD8+ T cells, T helper-1, Tc1, and NKT cells. The ratio of Tc1 to Tc2 cells increases significantly [111, 120, 125].

Following chemotherapy, both CD4+CD45RO+ and CD8+CD45RO+ memory T cells increased significantly and peaked around 2 weeks. The increase of memory T cells in ovarian cancer patients following chemotherapy probably opens the door of opportunity to develop long-term immunological memory, which could prevent recurrence and metastases [120]. Administration of dose-dense therapy, given weekly may hence be beneficial immunologically to mount an adequate immune response, compared to standard 3-week therapy. In addition, administration of immunotherapy agents may also be ideal during the period of 2 weeks after chemotherapy, where temporary immune system reconstitution takes place.

## 8. Clinical Studies

In the past two decades, paclitaxel had been administered in doses ranging from 60 to 250 mg/m^2^, over 1–96 hours and from 1 to 3 weekly intervals. Prolonged exposure up to 96 hours was believed to be effective as demonstrated with preclinical data but later refuted in large prospective studies [14, 126, 127]. A 24-hour infusion of paclitaxel was subsequently administered to reduce the risk of hypersensitivity reactions; however, it was shown to increase mucosal and bone marrow toxicity without improved efficacy [127, 128]. Development of effective premedication has led to administration of shorter infusions (<3 hours), which are better tolerated in particular, from a haematologic perspective, but patients remain at risk of myalgia and neuropathy at higher doses. Weekly one-hour low dose paclitaxel, also known as dose-dense therapy, has recently emerged as another possible strategy to improve outcomes.

## 9. Dose-Dense Therapy

Dose-dense treatment is defined by delivery of a maintained per-cycle dose or overall dose with administration of drug at shorter time intervals than is standard. This differs from dose intensity, which refers to dose (per unit weight or body surface area) delivered per unit time. Hence, either escalation of dose per cycle or reducing the time in between cycles may modify dose intensity [129, 130].

The rationale for the dose-dense approach of weekly paclitaxel is that more frequent delivery of moderate doses may achieve greater efficacy by allowing sustained exposure of dividing tumour cells to paclitaxel's cytotoxic and antiangiogenic effects, hence reducing resistance, delaying relapse, and increasing the possibility of cancer remission. Three 1-hour infusions of paclitaxel given at a week interval achieved greater dose-density as well as dose-intensity than a single administration every 3 weeks. The initial tissue concentration (>50 nM) for 1-hour infusion is similar to that of 3-hour infusion, but the concentration in the plasma, instead of lasting for 8 hours in a 3-hour infusion, rapidly falls below 50 nM in less than 75 minutes for 1-hour infusion and hence reduces the risk of neutropenia. However, the rate of fall experienced in tissue concentration of the drug is slower than plasma concentration of the drug; hence, efficacy is maintained [131, 132]. A shorter infusion time allows better control of toxicities, improved patient comfort, and reduced hospitalisation hours.

Several studies have used dose-dense paclitaxel in the treatment of advanced ovarian cancer and are summarized in Table 1. Based on phase 1 studies, the recommended dose for weekly paclitaxel ranges between 80 and 90 mg/m^2^, compared with 45 mg/m^2^ per week with 3-weekly paclitaxel, and is given over 1-hour duration. Weekly scheduling has demonstrated consistent activities in ovarian cancer studies [11–13]. Response rates were in the 20–50% range, where usually less than 10% would be expected. Grade-4 dose limiting toxicities, in particular neutropenia, were not evident at any dose below 100 mg/m^2^/week [11, 133].

Numerous phase II studies have explored the tolerability and the activity of weekly paclitaxel, either as a single agent or in combination with several other drugs or biological agents (Table 1). A number of regimens combining weekly paclitaxel with carboplatin have been used as first-line treatment in ovarian cancer. Carboplatin has been administered weekly at AUC of 2 with dose escalation in some studies, while paclitaxel had been infused at 60–80 mg/m^2^/week. This combined regimen was well tolerated with a low incidence of bone marrow suppression. Overall response rates have been high, in the range of 60–80% in most studies, even in heavily pretreated patients with platinum-resistant disease (disease recurrence or progression within 6 months of the last line of platinum-based therapy) [15–20, 134]. The promising data from these phase II trials then led to the evolution of larger phase III trials.

Three phase III studies, Japanese Gynecologic Oncology Group- (JGOG-) Trial 3016 [21–23], Multicentre Italian Trial in Ovarian Cancer-Trial 7 (MITO-7) [25], and Gynecologic Oncology Group GOG-262 Trial [24], have explored dose dense therapy. The results of these trials are summarised in Table 2.

The Japanese Gynecologic Oncology Group (JGOG) conducted a randomized phase 3 trial of dose-dense weekly paclitaxel, 80 mg/m^2^/week in combination with 3-weekly carboplatin compared to standard 3-weekly carboplatin and paclitaxel 180 mg/m^2^ in patients with advanced epithelial ovarian, peritoneal or fallopian tube cancer (JGOG 3016, NOVEL trial). In both arms, carboplatin was given at AUC 6 and the median number of cycles was six. In this large study (*n* = 637) with a median follow up of 77 months, there was an 11-month improvement in the median progression free survival (PFS) in the dose-dense treatment group compared with the standard treatment arm (28.2 versus 17.5 months, HR 0.76, 95% CI 0.62–0.91, *p* = 0.0037). Median OS was also higher in the dose-dense treatment arm (100.5 versus 62.2 months, HR 0.79, 95% CI 0.63–0.99, *p* = 0.039). On subgroup analysis, suboptimally debulked patients with residual disease of >1 cm (*n* = 342) had a significantly higher median overall survival in the dose dense arm (51.2 versus 33.5 months, HR-0.75, 95% CI 0.57–0.97, *p* = 0.027). There was no significant advantage to dose-dense treatment for patients with optimally cytoreduced disease. Dose-dense therapy survival benefits were also not seen in patients with clear cell or mucinous histology type, unlike serous type. However, patient treated with dose dense therapy had a significantly higher frequency of hematologic toxicity than with the standard arm (21.2% versus 9.4%), which resulted in lower treatment completion rates (63% versus 48%) with frequent episodes of delay (76% versus 67%) and dose reductions (48% versus 35%) [21, 22]. There was no significant difference in terms of overall quality of life between the two arms (*p* = 0.46) [23].

Using a similar regimen as in JGOG-3016, a Gynecologic Oncology Group (GOG)-262 Trial was conducted in the United States. In this group, most patients had stage III disease (66–70%) and two-thirds had suboptimal debulking with residual disease of >1 cm. This was a randomized phase III trial of 3 weekly paclitaxel versus dose-dense weekly paclitaxel in combination with carboplatin in patients with advanced epithelial ovarian, peritoneal, or fallopian tube cancer. Though optional, concurrent and maintenance bevacizumab were received by the majority of patients in both arms (83.5 versus 84.1%, resp.). There was no significant difference in PFS in the patients who received the dose dense paclitaxel/carboplatin regimen compared to those who received the standard paclitaxel/carboplatin regimen (14.8 versus 14.3 months, HR 0.97, 95% CI 0.79–1.18). However, in a subgroup analysis, there was a significant difference in PFS in the dose dense paclitaxel arm who did not receive bevacizumab, *n* = 112 (14.2 versus 10.3 months, HR 0.59, 95% CI 0.37–0.96, *p* = 0.003). Overall survival data is still immature. There was higher frequency of grade 3 anaemia (40.8 versus 15.7%, *p* < 0.001) and grade 2 sensory neuropathy (25.9 versus 17.8%, *p* = 0.012) but a lower incidence of neutropenia (72 versus 83%, *p* < 0.001) [24].

In 2008, the Multicentre Italian Trials in Ovarian Cancer (MITO-7) randomised 810 women in a phase 3 trial to receive either standard carboplatin (AUC = 6) and paclitaxel 175 mg/m^2^ every 3 weeks or a weekly schedule of paclitaxel (60 mg/m^2^) with carboplatin (AUC = 2). The majority of patients in both arms (83–90%) received six cycles of therapy. Most patients (58–63%) had stage II1 disease, 23% of them were suboptimally debulked. Median PFS did not differ significantly (17.3 months and 18.3 months; *p* = 0.66) between the standard and dose-dense arms, respectively. There was no significant difference in response rate between the two arms nor the estimated 2-year overall survival. Subgroup analysis did not identify any subgroup that may have benefited from the dose-dense regimen. Unlike the JGOG-3016 trial, the incidence of grade 3-4 neutropenia was significantly lower in the dose-dense group compared to the 3-weekly regimen (42% versus 50%). A similar pattern was seen for the incidence of febrile neutropenia (<1% versus 3%, *p* = 0.02), thrombocytopenia (1% versus 7%, *p* < 0.001), and other nonhaematological toxicities such as alopecia, vomiting, renal dysfunction, and neuropathy. Quality of life was reported to be significantly better in the dose-dense arm (*p* < 0.0001), which was discordant with the JGOG-3016 quality of life data [25].

In summary, there was significant improvement in median PFS and overall survival in studies using paclitaxel at 80 mg/m^2^/week dose-dense therapy [21, 24], but with higher haematological toxicity, whereas MITO-7 [25] which was using paclitaxel at 60 mg/m^2^/week was only equivalently effective but less toxic. This does not conclude that the higher paclitaxel dose is more clinically beneficial as there are many other confounding factors in all three studies.

In a multivariate analysis, the JGOG-3016 study [21] reported that a lower dose intensity of paclitaxel (<80%) was associated with poorer overall survival (hazard ratio = 1.42, 95% confidence interval 1.12–1.81, *p* = 0.004); however, it must be highlighted that the investigational arm carboplatin schedules were not standardised in these studies. The JGOG and GOG trials delivered a 3-weekly carboplatin (AUC = 6) arm, whereas in the MITO-7 study, the carboplatin was dose-dense (AUC 2 weekly). Although it is possible that the dose scheduling for the platinum-based agent is not essential for a survival benefit as shown by previous studies using dose-dense cisplatin, direct cross-trial comparison cannot be made. Unlike paclitaxel, carboplatin probably requires peak dose for optimal therapeutic benefit; hence, this may account for the inferior survival outcome in MITO-7 trial. Another confounding factor may be difference in ethnicity. All three studies used different ethnic populations and Asian patients in JGOG-3016 appear to have better survival outcomes than the MITO-7 Western population. Ethnicity, with possible underlying genetic causes, may be responsible for the variable responses to chemotherapy across individuals [135]. This was explored by Millward et al. who studied 68 Caucasian and Asian lung cancer patients treated with a combination of paclitaxel and carboplatin and found that the incidence of febrile neutropenia was 50% of an initially treated cohort of Asians and when carboplatin was subsequently dose reduced in all Asian patients, the overall incidence of febrile neutropenia was also reduced to 26% [136].

Although early clinical data from phase I and phase II dose-dense paclitaxel trials in advanced ovarian cancer were promising, the results from phase III studies in have been inconsistent as a result of differences in paclitaxel dose, carboplatin AUC and schedule, addition of bevacizumab as well as different ethnic populations. The results of a recently completed randomised three arms Gynaecologic Cancer InterGroup (GCIG) phase II trial, ICON 8 may further inform the clinical benefit of dose dense paclitaxel therapy.

This multicentre trial, involving 111 centres in the UK, South Korea, Mexico, Ireland, Australia, and New Zealand with a target population of 1485 patients with stage IC-IV epithelial ovarian cancer or peritoneal or fallopian tube cancer compared two dose-dense arms (i) JGOG-like arm: carboplatin AUC 5 plus weekly 80 mg/m^2^ paclitaxel and (ii) MITO-7-like arm: weekly carboplatin AUC 1.67 plus weekly 80 mg/m^2^ paclitaxel to the standard 3-weekly treatment (carboplatin AUC 5 plus 175 mg/m^2^ paclitaxel) for stage IC to IV epithelial ovarian cancer. The dose-dense arms of MITO-7 and JGOG-3016 as well as the ethnic heterogeneity will be further addressed.

## 10. Intraperitoneal (IP) Chemotherapy

In addition to dose and scheduling, the route of administration of chemotherapy agents plays an important role in outcome. Intraperitoneal spread is common and likely an early event in epithelial ovarian cancer; hence the delivery of chemotherapy intraperitoneally in theory would expose the microcirculation of tumour to higher doses of drug than systemic administration, while limiting systemic toxicity. The ideal drug for IP chemotherapy is one that is systemically effective, penetrates deep into the inner core of tumour tissue, and remains within the peritoneal cavity for a prolonged period of time [137–139].

Early clinical studies confirmed that indeed the peritoneal cavity could be exposed to 10 to 20-fold higher (cisplatin and carboplatin) drug concentration than when using the intravenous approach and since 1978 this route had been adopted as a form of therapeutic intervention [137, 139]. A subsequent phase I trial showed that IP paclitaxel had dose-limiting toxicity in the form of abdominal pain [140]. Another phase 1 GOG pilot study administered weekly IP paclitaxel at an initial dose of 20 mg/m^2^ for 16 weeks to 33 patients and found that IP paclitaxel at this dose was feasible and well tolerated. At dose levels 60 to 65 mg/m^2^, there were significant levels of paclitaxel for a week after drug administration, suggesting very slow clearance and continuous exposure of the peritoneal cavity to active drug concentrations [132].

Hence, this initiated a phase II study [141] that used paclitaxel intraperitoneally at a dose of 60–65 mg/m^2^/week. In this phase II GOG study, 76 patients with small-volume residual disease carcinoma (0.5 cm or less) involving the ovary, peritoneal, or fallopian tube were treated with paclitaxel 60 mg/m^2^/week intraperitoneally for 16 weeks followed by surgical evaluation in patients without disease progression. The Gynaecologic Oncology group (GOG) currently defines no visible disease as microscopic disease, being the ideal surgical outcome. 75% of patients had received prior systemic therapy and 70% received all 16 courses that were planned. Of 28 patients with microscopic disease at the start of therapy, 61% achieved a surgically defined complete response, whereas only 1 out of 31 patients with macroscopic disease achieved a complete response. The median time-to-recurrence was 16.7 months with an estimated survival rate at 2 years being 58%. Treatment was generally well tolerated [141].

Another study looked into the effect of IP paclitaxel as consolidation therapy in advanced ovarian cancer. Twenty-eight patients who had initial residual macroscopic disease (<1 cm) after primary surgery or interval debulking but had complete pathological response following standard treatment with at least 6 cycles of platinum-based chemotherapy regimen, received paclitaxel 60 mg/m^2^/week intraperitoneally for 12–16 weeks as consolidation therapy. The median time to recurrence was 25 months but overall survival did not differ significantly when compared with control group submitted to observation only. Treatment-related toxicity was mild and technical difficulties were observed in 11% of patients [142].

Multicenter randomised phase 3 trials as well as meta-analysis of IP chemotherapy have been performed and demonstrated that chemotherapy drugs administered intraperitoneally are superior to intravenous standard regimens in patient with optimally debulked epithelial ovarian cancer. The most impressive data comes from GOG 114 [14] and 172 trial [143]. In GOG 114, patients received either six cycles of intravenous paclitaxel 135 mg/m^2^ and cisplatin 75 mg/m^2^ every 3 weeks or intravenous carboplatin (AUC = 9) every 28 days for two cycles, followed by six 3-weekly cycles of intravenous paclitaxel 135 mg/m^2^ and IP cisplatin 100 mg/m^2^ [14], whereas in GOG 172, patients received either intravenous paclitaxel 135 mg/m^2^ followed by intravenous cisplatin 75 mg/m^2^ or intravenous paclitaxel 135 mg/m^2^ followed by IP combination of cisplatin 100 mg/m^2^ and paclitaxel 60 mg/m^2^ on day 8. Both trials had a combined total of 876 women with stage III epithelial ovarian carcinoma, optimally debulked to ≤1 cm residual tumour [143]. The median follow-up for GOG 114 [14] was 13.8 years with GOG 172 [143] was 9.7 years. The primary results of the individual trials reported up to 6-month improvement in median PFS following IP therapy. In the combined analysis of the data from both trials, the data continued to show a 5-month difference in favour of IP therapy (25 versus 20 months), which translates into 16% reduction in the hazard ratio for progression (*p* = 0.03). In GOG 114 [14] reported in 2001, there was only borderline improvement in overall survival associated with this regimen (median, 63 versus 52 months; relative risk, 0.81; *p* = 0.05).

However, in combined analysis in 2013 which was presented at the Society of Gynecologic Oncology Annual Meeting [144], the overall survival showed an 11-month improvement in favour of IP therapy in GOG 114 whereas GOG 172 reported a 16-month survival advantage. Among patients treated with IP therapy, the 5-year survival rate increased from 18% following completion of 1-2 cycles to 59% for patients who completed 5-6 cycles of treatment. However, there was increased toxicity observed in the IP arm; grade 3-4 leukopenia, thrombocytopenia, and abdominal pain. Only 42% of patients in the GOG172 trial [143] completed all six cycles of planned treatment in the IP arm, while 18% of patients in GOG 114 trial [14] received fewer than two courses of IP chemotherapy.

Another randomised phase III trial involving patients with stages II-III epithelial ovarian carcinoma is the GOG 252 study, with three arms: Arm 1-dose-dense intravenous carboplatin and paclitaxel, Arm 2-dose-dense intravenous paclitaxel and IP carboplatin, and Arm 3 consists of 3-weekly intravenous paclitaxel (day 1) followed by IP cisplatin (day 2) and IP paclitaxel (day 3) regimen. All three arms also incorporate the combination and maintenance of bevacizumab. GOG 252 has completed accrual and results are awaited [139].

## 11. Newer Forms of Paclitaxel

Development of next-generation taxanes has taken place over the past 40 years aimed at eliminating toxicity, improving efficacy and ease of administration. Abraxane is a novel compound which incorporates paclitaxel into an albumin nanoparticle, soluble in saline, hence eliminating the need for Cremophor EL which is responsible for hypersensitivity reactions experienced during paclitaxel infusion [145]. The nanoparticle albumin in nab-paclitaxel is able to bind to glycoprotein gp60 receptor, an albumin receptor, and activate caveolin-1, a protein that in human is encoded by CAV-1 gene, leading to formation of caveoli, hence allowing nab-paclitaxel to migrate across the endothelial cell membrane into the interstitial space leading to higher intratumoural drug concentration. In addition, Abraxane exhibits linear pharmacokinetics and can therefore be given at a relatively higher dose than standard intravenous paclitaxel, leading to a higher therapeutic ratio and heightened efficacy in solid tumours, mainly metastatic breast cancer [146, 147]. Nab-paclitaxel had FDA approval in 2005 for treatment of breast cancer [148].

Nab-paclitaxel has been studied in recurrent ovarian cancer. As a single agent in phase II, 44 patients with recurrent ovarian cancer were treated with Nab-paclitaxel 260 mg/m^2^ intravenously, for 30 minutes every 3 weeks for 6 cycles. Ninety-two percent of patients were platinum-sensitive, while 89% had had previous exposure to standard taxane. The objective response rate was 64% with 15 patients achieving complete response and 13 of them had partial response. Estimated median PFS was 8.5 months. No hypersensitivity reactions were observed and there were infrequent cases of grade 4 neutropenia (11%) and grade 2-3 neuropathy (13%) [149].

Another phase II study by GOG enrolled 51 patients with platinum- and taxane-resistant recurrent ovarian cancer, out of which 47 were evaluable. The objective response rate was 38%, with one patient achieving a complete response and 10 patients a partial response. The median PFS was 4.5 months while overall survival rate was 17.4 months. Severe haematological and nonhaematological toxicities including neurotoxicity were uncommon. These results are quite impressive given the treatment population consisted of patients with very poor prognosis with a median platinum and taxane free interval of 21 days and 70% had their recurrence within 3 months of completion of primary treatment [150]. Several other trials of this formulation are in progress.

Paclitaxel poliglumex (PPX), also known as Xyotax, is another prodrug, a novel conjugate of paclitaxel and *α*-poly-L-glutamic acid, which accumulates within tumour tissue due to increased permeability of the tumour vessels and lack of lymphatic drainage [148, 151, 152]. Paclitaxel poliglumex enhances solubility of paclitaxel, allows direct delivery to the intratumoural microenvironment and allows prolonged exposure to the active drug while minimising systemic toxicities [151]. In Phase I dose escalation studies as a single agent, the recommended dose of PPX was 235 mg/m^2^ over 10 minutes every 3 weeks or 70 mg/m^2^ weekly [153]. In a Phase II GOG study of relapsed ovarian cancer, a response rate of 16% was seen, with infrequent serious adverse events, grade 3 and 4 neutropenia (24% and 20%), and grade 3 neuropathy (24%) [154]. A phase III trial (GOG-0212) on the use of PPX as consolidation or maintenance therapy in advanced ovarian cancer is in progress.

DHA-paclitaxel, also known as Taxoprexin is a prodrug where paclitaxel is covalently conjugated with the naturally occurring omega-3 fatty acid docosahexaenoic acid (DHA), a fatty acid that is easily taken up by tumour cells, hence increasing intratumoural concentration of paclitaxel leading tumour cell apoptosis [155, 156]. DHA-paclitaxel exhibits linear pharmacokinetics and lower toxicity and also ease of administration when compared to conventional paclitaxel and has demonstrated antineoplastic activity in animal models of cancer as well as in a phase III trial involving metastatic melanoma [156–160]. In a recent study on human ovarian cancer cells it was found that DHA could reverse paclitaxel resistance by inhibiting P-gp as well as downregulating the expression of multidrug resistance associated proteins (MRP) and inhibiting the activity of NF-*κ*B and p38 MAPK signalling pathways [161].

## 12. Future Directions

A plateau has been reached regarding the benefits associated with standard 3-weekly intravenous administration of cytotoxic chemotherapy in advanced epithelial ovarian cancer. The ongoing battle for a better treatment regimen to delay relapse, promote remission, and most importantly improve the quality of life of patients with advanced epithelial ovarian cancer need not to go further than the ability to relook at the chemotherapy agents that are already available in a different approach.

Novel compounds to date have not yet shown clinical superiority, and parent compounds such as paclitaxel continue to surprise us with their feasibility, effectiveness, and manageable toxicity profiles. Intraperitoneal paclitaxel will require further evaluation. Better effort in understanding the mechanism of action of drugs including the role of dose scheduling and the effect on the immune system may provide a more cost-effective route to better clinical outcomes.

## Figures and Tables

**Table 1:** Phases I and II studies on intravenous dose-dense paclitaxel.

Study	Regimen		*n* (#)	PI (m)	RR (%)	CR (*n*)	Median PFS (m)	Median OS (m)	Adverse effects	Comments
	Paclitaxel dose (mg/m^2^)	Carboplatin dose (mg/m^2^)								
Phase I: single agent										
Lofflerr et al., 1996 [11]	40–90/week 1-hour infusion for 6 weeks		50		40	5	NA	NA	(i) Hematologic toxicity-mild (ii) No grade 3 or 4 haematological toxicity, neurological or cardiotoxicity up to 90 mg/m^2^/wk. (iii) No hypersensitivity reactions (patients received premedication).	(i) 100% prior chemotherapy. (ii) Median dose intensity was 410 mg/m^2^/6 wk (range, 200 to 540 mg/m^2^/6 wk).
Fennelly et al., 1997 [12]	40–100/week		18		30	NA	NA	NA	(i) No mucositis or grade III neuropathy was seen. (ii) Alopecia occurred in one.	(i) 100% prior chemotherapy (2–5 prior regimens). (ii) Partial responses were seen in four of 13 assessable patients (30%). (iii) Dose-limiting toxicity was reached at 100 mg/m^2^. (iv) 2-3 patients experience treatment delay. (v) Maximum-tolerated dose of 80 mg/m^2^.
Abu-Rustum et al., 1997 [13]	60–100 over 1 hour/week		45		28.9	NA	NA	NA	(i) No patient had febrile neutropenia.	(i) 100% prior chemotherapy (1–8 prior regimens).

Phase II: Single agent										
Markman et al., 2001 [14]	80 over 1 hour/week		53 (52)		25	NA	NA	NA	Therapy discontinued in (i) 4 patients who had peripheral neuropathy, (ii) 1 patient due to painful fingernail beds.	(i) 100% platinum and paclitaxel resistant. (ii) Therapy was discontinued in 5 patients because of toxicity. (iii) Only 13 (1%) were modified (dose reduction or treatment delay) because of side effects.

Phase II: Combined with platinum agent										
Kikuchi et al., 2005 [15]	80 over 1 hour/week	AUC 2/week	27	>6	81	8	48.3	NA	(i) Neutropenia (1.7%), thrombocytopenia (5.1%). (ii) No cases of peripheral neuropathy (grades 3 and 4) in weekly combined T-C.	
Cadron et al., 2007 [16]	90 over 1 hour/week	AUC 4/week	24 9	>6 ≤6	73 38		NR 8	10.5 6.75	(i) Grade 3/4 neutropenia (34%) and neutropenic fever in 2%. (ii) Nausea and vomiting and fatigue were the most frequent nonhematological side effects.	(i) Dose reduction was necessary in 25% of patients.
Pignata et al., 2008 [17]	60 over 1 hour/week	AUC 2/week	24 (13)^∗∗^	1st line	38.5	2	32.0	13.6	(i) No toxic death was recorded. (ii) Grade 2 anaemia was reported in seven patients (27%). (iii) No febrile neutropenia was observed. (iv) Grade 3 and 4 neutropenia was recorded in five (19%) and one (4%) patients. (v) Grade 3 thrombocytopenia occurred in one patient (4%).	
Safra et al., 2014 [18]	80 over 1 hour/week	AUC 2/week	133	1st line	86.4		64.5	27.4	(i) Higher anaemia (grade III + IV: 6.8% vs. 5.3%) + IV neutropenia (14.4% vs. 6.9%) decreased grade II alopecia (23.5% vs. 98.1%) and thrombocytopenia (grade III + IV: 0 vs. 2.3%).	
Havrilesky et al., 2003 [19]	80 over 1 hour/week	AUC 2/week D1, D8, D15 until progression/CR + 8 courses	29 21 8	Total group >6 <6	83 100 38	16 15 1	11.4	11.5 13.7 3.2	(i) Hematologic toxicity was common (grade 3 neutropenia 32%, no grade 4 neutropenia, grade 3 or 4 thrombocytopenia 14.2%).	(i) Toxicity managed by treatment delay, dose reduction of paclitaxel, or discontinuation of carboplatin.
van der Burg et al., 2014 [20]	90 over 1 hour/week	AUC 4/week D1, D8 D15 D29 D36 D49 + 6x TC q3w	108 65 43	Total group >6 <6	76 58	16 42	26 15	8 13	(i) Neutropenia 30%, thrombocytopenia 8%, febrile neutropenia 0.5%. (ii) Nonhaematologic toxicity was low.	(i) Treatment was delayed in 16%, and dose reduced in 2% of cycles.

*n*: number of patients, #: assessable for response, m: months, PI: platinum treatment free interval, RR: response rate, CR: complete remission, T: paclitaxel, C: carboplatin, PFS: progression free survival, OS: overall survival, NA: not available in paper or abstract, and NR: not yet reached.

^∗∗^Elderly population, patient aged ≥70 years.

**Table 2:** Phase III studies on intravenous dose-dense paclitaxel.

Study	Regimen	*n*	Median PFS (m)	Median OS (m)	Adverse effects	Comments
Katsumata et al., 2009 [21] Katsumata et al., 2013 [22] Harano et al., 2014 [23]	JGOG 3016	631			(i) Haematological toxicity higher in dose dense-21.1 versus 9.4%	Lower treatment completion in dose-dense 63 versus 48% Frequent episodes of delay in dose-dense 76 versus 67% Dose reductions in dose-dense 48 versus 35% No significant difference in overall QOL between both groups (*p* = 0.46)
	Paclitaxel 80 mg/m^2^ D1, 8, 15 + carboplatin AUC 6, 3 weekly versus Paclitaxel 180 mg/m^2^ + carboplatin AUC 6, 3 weekly	312	28.2	100.5		
	Both given for 6–9 cycles	319	17.5	62.2		

Chan et al., 2013 [24]	GOG-262				(i) Higher frequency of grade 3 anaemia (40.8 versus 15.7%, *p* < 0.001), grade 2 sensory neuropathy (25.9 versus 17.8%, *p* = 0.012) but lower incidence of neutropenia (72 versus 83%, *p* < 0.001)
	Paclitaxel 80 mg/m^2^ D1, 8, 15 + carboplatin AUC 6, 3 weekly versus Paclitaxel 175 mg/m^2^ + carboplatin AUC 6, 3 weekly	346	14.8	NR	
	Both given for 6 cycles	346	14.3	NR	
	Bevacizumab given optionally	112 (did not receive bevazicumab)	14.2 vs 10.3		

Pignata et al., 2014 [25]	MITO-7	810			(i) Lower incidence of grade 3-4 neutropenia (42 versus 50%), febrile neutropenia (<1 versus 3%, *p* = 0.02), thrombocytopenia (1 versus 7%, *p* < 0.001), and other nonhaematological toxicities such as alopecia, vomiting, renal dysfunction, and neuropathy	Quality of life better in dose dense arm (*p* < 0.0001)
	Paclitaxel 60 mg/m^2^, weekly + carboplatin AUC 2, weekly versus Paclitaxel 175 mg/m^2^ + carboplatin AUC 6, 3 weekly	406 404	18.3 17.3	77^∗^ 79^∗^		

*n*: number of patients, m: months, D: day PFS: progression free survival, OS: overall survival, NA: not available in paper or abstract, and NR: not yet reached.

^∗^Survival rate at 2 years.
